# Elucidating Cold-Stress-Induced Metabolic and Transcriptional Reprogramming in *Tuta absoluta* Larvae Through Integrated Multi-Omics Analysis

**DOI:** 10.3390/biology15151308

**Published:** 2026-08-05

**Authors:** Bo Feng, Chuanhong Feng, Zhihao Ling, Liping Xiong, Xi Yang, Jiatao Huang, Hangtian Zhou, Tao Hu, Lingzhi Huang, Yong Yin, Kaidi Zheng

**Affiliations:** 1 Mianyang Teachers College, College of Biology and Pharmaceutical Sciences, Mianyang 621000, China; fb820528@aliyun.com (B.F.); x2016766@163.com (L.X.); 15282618441@163.com (X.Y.); 15700210683@163.com (J.H.); 15008359286@163.com (H.Z.); 2 Plant Protection Station of Sichuan Provincial Department of Agriculture and Rural Affairs, Chengdu 610000, China; fengchuanhong8@163.com (C.F.); cqhutaozjjt@163.com (T.H.); huanglingzhi0421@163.com (L.H.); 3 Chengdu Genepre Technology Co., Ltd., Chengdu 610095, China; lingzhihao@genepre.com

**Keywords:** *Tuta absoluta*, temperature stress, larval development, oxidative stress, energy metabolism, metabolomics, transcriptomics

## Abstract

Exposure to stressful low temperatures during development can cause chilling injury, leading to impaired physiological performance. In insects, chilling injury is often associated with metabolic imbalance, oxidative stress and disruption of energy homeostasis, which can collectively compromise survival and growth. Because *Tuta absoluta* (Meyrick, 1917) is a tomato pest adapted to warm environments, we hypothesized that low-temperature exposure would induce chilling injury by disrupting metabolism and cellular function. We investigated the responses of *T. absoluta* larvae to three thermal regimes (25, 15 and 5 °C) over a 7-day period, using integrated physiological, metabolomic and transcriptomic analysis. Low-temperature stress reduced survival and feeding performance, accompanied by suppressed digestive enzyme activities (α-amylase, lipase and trypsin) and depletion of glycogen reserves, indicating impaired energy acquisition. In contrast, increased trehalose and proline accumulation suggested a shift toward protective metabolism. Importantly, low temperature induced a pronounced decoupling of energy metabolism and redox homeostasis, characterized by reduced antioxidant capacity (peroxidase; POD and superoxide dismutase; SOD) and elevated levels of reactive oxygen species (ROS). Metabolomic and transcriptomic analysis of stressed larvae revealed alterations in amino acid and carbohydrate metabolism, showing differential regulation of the genes involved in energy production, oxidative stress responses and growth. Integrative analysis demonstrated that metabolic reprogramming and transcriptional regulation are tightly linked under low-temperature conditions, revealing a resource allocation trade-off between growth and stress defense. Together, these findings identify metabolic and redox imbalances as mechanisms underlying cold-induced physiological decline, providing new insight into how low temperature constrains insect performance.

## Simple Summary

Low temperatures can strongly affect insect development, survival and physiological performance, particularly in species adapted to warm environments. Here, we characterized the responses of *Tuta absoluta* larvae after continuous exposure to three thermal regimes—25, 15 and 5 °C—for 7 days using integrated physiological, metabolomic and transcriptomic analyses. Our study shows that cold exposure disrupts feeding, digestive enzyme activity, energy storage and redox balance in *T. absoluta* larvae. Larvae exposed to lower temperatures showed reduced survival and growth performance, together with depleted glycogen reserves and weakened antioxidant defenses. Meanwhile, protective compounds such as trehalose and proline accumulated, suggesting a shift from normal growth metabolism toward stress protection. Molecular analyses further revealed changes in pathways associated with carbohydrate and amino acid metabolism, energy production, oxidative stress, and development. Overall, the findings indicate that cold stress forces *T. absoluta* larvae to reallocate resources from growth and feeding toward survival. This work provides useful insight into the physiological and molecular basis of low-temperature sensitivity in an important agricultural pest.

## 1. Introduction

Among invasive lepidopteran pests, the tomato leafminer, *Tuta absoluta* (Meyrick, 1917), stands out as a globally significant threat to solanaceous crops, especially tomato production [1,2,3]. Since its rapid expansion from South America to Europe, Africa and Asia, this species has demonstrated remarkable ecological adaptability, enabling it to establish and persist across diverse climatic zones. While its invasive success has often been attributed to its reproductive capacity and cryptic feeding behavior, physiological plasticity is increasingly recognized as a key contributing factor. In particular, the ability of this pest to rapidly colonize new geographic regions and establish stable populations depends heavily on its capacity to tolerate and adapt to local environmental conditions, especially temperature [4,5]. Understanding the fundamental biology and physiological responses of *T. absoluta* to environmental stressors, especially cold stress [6], is critical for developing effective and sustainable pest control strategies and for predicting a pest’s potential geographic expansion. Although both high- and low-temperature extremes influence insect distribution and performance, this study specifically focuses on prolonged low-temperature exposure because a cold limitation represents a critical environmental constraint affecting the seasonal persistence and establishment potential of invasive insects in temperate regions.

Temperature is among the most influential environmental variables governing insect physiology, development and geographic distribution [7,8]. For a warm-climate-associated invasive pest of tropical origin such as *T. absoluta*, temperature acts as both a determinant of reproductive success in favorable environments and a potential limiting factor in regions characterized by seasonal temperature fluctuations [9]. As global climate patterns shift and these herbivores continue to expand their geographic range into temperate regions, the following critical knowledge gap emerges: how *T. absoluta* responds physiologically and developmentally to the range of temperatures encountered across its expanding distribution, particularly at cold extremes. Addressing this question is essential for predicting where *T. absoluta* can establish sustainable populations, how seasonal population dynamics will unfold and whether temperature-based management strategies can exploit this pest’s thermal vulnerabilities [10,11,12]. The current understanding of *T. absoluta*’s thermal biology is fragmented, focusing primarily on developmental thresholds and optimal temperatures while neglecting comprehensive characterization of low-temperature stress responses across the physiological, metabolic and molecular dimensions.

Low temperatures can directly affect insect physiological processes and may also indirectly influence herbivores through temperature-induced changes in host plant quality and nutrient availability. These combined effects can alter nutrient assimilation and internal energy allocation, forcing insects to rely on metabolic flexibility to sustain essential functions [13,14]. When exposed to low temperatures, insects redirect their metabolic pathways to prioritize survival over growth, leading to dynamic changes in energy metabolism and resource allocation [15]. Such metabolic reprogramming is particularly important during immature stages, when energy demands are higher and developmental processes are tightly regulated [16]. The synthesis and accumulation of protective compounds, alterations in nutritional processing, reorganization of protein pools and activation of cellular defense mechanisms represent critical adaptive responses [17]. Additionally, low temperatures can interfere with mitochondrial electron transport and cellular redox reactions, leading to the accumulation of reactive oxygen species (ROS), which pose a serious threat to cellular integrity [18,19]. To mitigate these risks, insects employ antioxidant defense systems that neutralize ROS and preserve redox stability. The efficiency of these defenses is often a key determinant of stress tolerance and recovery capacity [20,21]. Importantly, oxidative stress responses are not independent of metabolism; instead, they are tightly coupled with energy regulation, overall physiological condition and, potentially, the nutritional environment experienced by herbivorous insects within host tissues.

Recent advances in systems biology have highlighted that insect responses to environmental stress are underpinned by coordinated changes at the molecular level [22,23]. Rather than relying on adjustment of a single pathway, insects typically activate broad regulatory networks that alter gene expression patterns and downstream metabolic processes [23]. High-throughput omics approaches have revealed that low-temperature stress can reshape the entire metabolic landscape, affecting pathways related to energy production, stress protection and cellular maintenance [22]. These molecular adjustments provide the mechanistic basis for the observed phenotypic and physiological responses, yet they remain insufficiently characterized for several agriculturally important pests, especially during the larval stages of endophagous species such as *T. absoluta*. The integration of metabolomics and transcriptomic data has proven particularly powerful for uncovering stress adaptation strategies in insects [24]. Despite their potential, such integrative frameworks have rarely been applied to invasive pest species facing thermal stress, leaving critical gaps in our understanding of how these organisms cope with environmental constraints.

Larval stages play a critical role in determining insect sensitivity and resilience to environmental stress [25,26,27]. As the primary feeding stage responsible for crop damage, larvae experience direct exposure to environmental fluctuations while simultaneously supporting rapid growth and tissue differentiation. Therefore, elucidating larval responses to cold stress provides valuable insights into both population regulation and pest impacts under variable climatic conditions [28]. Despite the economic importance of *T. absoluta*, existing studies have largely focused on its ecology, control strategies and thermal performance under different temperature conditions [1,29,30]. In contrast, the physiological and molecular consequences of cold exposure, particularly at the larval stage, remain poorly understood. Several studies have investigated the transcriptional or biochemical responses to thermal stress; however, integrated approaches connecting molecular changes with organism-level physiological performance remain limited [22].

The present study focuses exclusively on the larval stage of *T. absoluta*, the most destructive and metabolically active life stage responsible for crop damage. Larvae are the primary target of pest control strategies and are highly sensitive to temperature-dependent physiological constraints [31,32]. Rather than focusing on isolated traits, we aimed to examine how cold exposure reshapes larval performance through coordinated physiological, metabolic and molecular adjustments. By combining phenotypic observations with system-level analyses, this approach enables a more comprehensive understanding of how larvae cope with thermal constraints. We hypothesized that cold stress would trigger a graded, temperature-dependent response in *T. absoluta* larvae, characterized by coordinated changes in developmental regulation, metabolic organization, oxidative balance and gene expression. This research provides new mechanistic insights into cold stress adaptation in *T. absoluta* and contributes to a broader understanding of thermal resilience in invasive insect pests under changing climatic conditions.

## 2. Materials and Methods

### 2.1. Plant Material and Insect Culture

Tomato plants (*S. lycopersicum*, cv. ‘Heinz’) were grown from surface-sterilized seeds and disinfected in 1% sodium-hypochlorite (NaClO) solution for 5 min, followed by three thorough rinses with sterile distilled water. The sterilized seeds were placed on moistened filter paper in Petri dishes (8.5 cm width) and allowed to germinate. Once small seedlings (a height of 2–3 cm) emerged, plants were transferred into small plastic pots (115 mm × 110 mm) filled with sterilized peat-based potting soil (Shenzhen Shenglvyuan Horticulture Co., Ltd., Shenzhen, China). Tomato plants were then grown in a glasshouse under controlled conditions of 24 ± 2 °C, 16:08 h light/dark cycles with a light intensity of 600–800 μmol m^−2^ s^−1^ (600 W, Lucagrow) and were watered regularly. No additional fertilizer was applied during plant growth, and plants were maintained using only the nutrient availability provided by the peat-based potting soil. Tomato plants were grown for 25 days under controlled glasshouse conditions before leaflets were collected for larval feeding assays.

A laboratory colony of the tomato leafminer, *Tuta absoluta* (Meyrick, 1917), was maintained on potted tomato plants (*Solanum lycopersicum*, *cv*. Heinz) under controlled environmental conditions. *T. absoluta* specimens were obtained from Miyi City, Sichuan Province, China, and their population was reared for more than 10 generations on tomato plants. Insects were reared in a controlled environment chamber at 24 ± 2 °C, 16 h:8 h light/dark cycles and relative humidity of 60–70%. For experimental assays, adult females of *T. absoluta* from the stock cultures were introduced onto healthy plants and allowed to oviposit freely. Fully developed third-instar larvae were collected and randomly assigned to temperature treatments. All the experiments were conducted in separate climate incubators (MLR-352-PE, Panasonic, Kadoma, Osaka, Japan) set to one of three temperature treatments, control temperature (CT, 25 °C), moderate cold stress (MCS, 15 °C), or severe cold stress (SCS, 5 °C), under 16 h:8 h light/dark cycles, light intensity 400 μmol m^−2^ s^−1^ (Mitsubishi tube light, 40 W). Larvae were individually fed fresh tomato leaves for 7 days (unless otherwise specified), and individuals subjected to low-temperature stress during this period were used for all biochemical, metabolomic and transcriptomic analyses. Prior to sampling, larvae were gently removed from tomato leaves and kept without food for 3 h under their respective temperature conditions to allow gut clearance and minimize interference from plant-derived compounds. Larvae were then thoroughly rinsed with distilled water, carefully blotted dry, immediately snap-frozen in liquid nitrogen and stored at −80 °C until further analyses. Unless otherwise stated, biological replicates were prepared independently from different larvae assigned to each temperature treatment.

### 2.2. Larval Thermal Biology Assays

Polyethylene containers (25 cm height × 20 cm width × 17 cm length) were placed in climate chambers maintained under the three temperature treatments—CT, MCS and SCS—and the environmental conditions described in Section 2.1. Larvae were provided with a fresh tomato leaflet, with the petiole wrapped in moistened cotton to avoid desiccation. Leaflets were surface sterilized by immersion in 1% NaClO solution, followed by thorough rinsing with distilled water prior to use. Larvae were monitored daily to record survival and developmental progressions. After the 7-day exposure period, surviving larvae were carefully collected from leaf tissues using a camel-hair brush (Dynasty B-900, FM Brush Co., Ltd., Lamphun, Thailand) and forceps (Texnet, Suzhou, China) and individually weighed using an analytical balance (Mettler Toledo GmbH, Greifensee, Switzerland) to determine fresh body weight. Larval feeding activity was quantified by measuring the leaf area mined by each larva using a desktop-connected oplenic digital camera (SMZ745T, Nikon, Tokyo, Japan) [33]. Each polyethylene container contained one third-instar larva and one fresh tomato leaflet.

Chill-coma recovery time (CCRT) of leafminer larvae was assessed following established protocols for insect cold tolerance assays, with slight modifications [34]. Third-instar larvae reared under different temperature regimes were individually placed into 2 mL microcentrifuge tubes (Biosharp Life Sciences, Hefei, Anhui, China) to prevent physical contact during chilling. Larvae were then transferred into a sealed zip-lock bag and submerged in a temperature-controlled water bath containing a 1:1 mixture of water and propylene glycol (Zhejiang polymer chemicals Co., Ltd. Hangzhou, Zhejiang, China), maintained at 0 °C. Larvae were held at this chill coma-inducing temperature for 1 h and then immediately removed and transferred to a climate chamber maintained at 24 ± 2 °C for recovery. CCRT was measured as the duration for each larva to regain coordinated movement after removal from cold treatment, and each larva represented one biological replicate, with 10 larvae examined per treatment.

### 2.3. Larval Digestive Physiology and Energy Metabolism

Digestive enzyme activities in the midguts of stressed larvae were determined using a previously established method with minor modifications for sample preparation [35]. Larvae from each treatment group were randomly selected, and midguts were carefully dissected in ice-cold distilled water to minimize enzymatic degradation. The dissected midgut tissues were immediately homogenized in an ice-cold buffer at a 1:1 (*w/v*) tissue–buffer ratio using a glass homogenizer. Homogenates were centrifuged at 12,000 rpm for 20 min at 4 °C, and the resulting supernatants were collected and stored at −20 °C until further analysis. Enzyme activities were quantified using commercial assay kits (Nanjing Jiancheng Bioengineering Institute, Nanjing, China) according to the manufacturer’s protocols (*n* = 5 tests/treatment). α-amylase activities (C016-1-1) were measured spectrophotometrically (Spectrophotometer 1780, Shimadzu, Kyoto, Japan) at 660 nm based on starch hydrolysis and expressed as units per milligram of protein (U/mg^−1^ protein), where one unit was defined as the enzyme amount required to hydrolyze 10 mg of starch within 30 min at 37 °C [36]. Lipase activity (A054-1-1) was determined colorimetrically by measuring the absorbance of the reaction product at 580 nm, which reflects the enzymatic hydrolysis of the lipase-specific substrate and expressed in U/mg^−1^ protein [37]. Trypsin-like protease activities (A080-2-2) were determined based on the absorbance of the reaction product measured at 253 nm following the hydrolysis of a trypsin-specific substrate and expressed as U/mg^−1^protein [38].

Total protein content (A045-3-2) was measured spectrophotometrically at 562 nm using the bicinchoninic acid method and used for normalization (*n* = 6 tests/treatment). Glycogen content (A043-1-1) was quantified spectrophotometrically at 620 nm following acid hydrolysis and anthrone reactions [39]. Trehalose content (A149-1-1) was determined from larval midgut extracts following extraction and centrifugation at 8000 rpm for 10 min, reaction with assay reagents and brief heating and quantification by measuring absorbance at 620 nm with values normalized to sample protein content [40]. Proline content was measured using a Solarbio assay kit (Cat# BC0295, Beijing, China) according to the manufacturer’s instructions. Larval tissues were homogenized, reacted with the kit reagents, heated and centrifuged, and absorbance was recorded at 520 nm (*n* = 5 replicates/treatment). For each enzyme assay, one biological replicate consisted of a pool of 10 independently dissected larval midguts.

### 2.4. Antioxidant Enzyme Activities Assays

Antioxidant enzyme activities were determined using commercial assay kits (Solarbio Life Sciences, Beijing, China). Larvae were homogenized in ice-cold 0.05 M phosphate buffer (pH 7.8) containing 0.1 mM EDTA (Thermo Fisher Scientific, Waltham, MA, USA) and 1% polyvinylpyrrolidone (Sigma-Aldrich, St. Louis, MO, USA) with a FastPrep^®^-24 Classic Instrument (MP Biomedicals, Washington, DC, USA), followed by centrifugation at 11,000 rpm for 10 min at 4 °C. The resulting supernatants were collected and stored at −70 °C until analysis (*n* = 5 tests/treatment).

Peroxidase (POD) activity (BC0095) was measured based on hydrogen peroxide (H_2_O_2_)-dependent substrate oxidation, with absorbance recorded at 420 nm and enzyme activity expressed relative to protein content at 37 °C. Superoxide dismutase (SOD) activity (BC0175) was determined using a total SOD assay kit employing the xanthine–xanthine oxidase method, in which inhibition of superoxide radical formation was quantified at 550 nm; one unit of SOD activity was defined as the amount of enzyme causing 50% inhibition of superoxide generation. Catalase (CAT) activity (BC0200) was measured based on H_2_O_2_ decomposition and ammonium molybdate ((NH_4_)_2_MoO_4_) complex formation, with absorbance measured at 405 nm and activity expressed per unit protein [41,42].

### 2.5. Confocal Microscopy

Reactive oxygen species (ROS) levels in larvae were assessed using the fluorescent probe 2′,7′-dichlorodihydrofluorescein diacetate (DCFH-DA) (Sigma-Aldrich, St. Louis, MO, USA) (*n* = 3 tests/treatment). Treated larvae were collected from leaves and washed with PBS three times and incubated with 50 µM DCFH-DA solution (whole larvae incubated) for 30 min at room temperature in the dark. After staining, samples were washed again three times with PBS to remove excess dye and immediately mounted on glass slides. Fluorescence imaging was performed using a Zeiss LSM confocal laser-scanning microscope (Carl Zeiss, LSM 980 Airyscan-2, Jena, Germany) with excitation at 488 nm and emission collected at 500–550 nm.

### 2.6. Metabolomics Analysis

#### 2.6.1. Sample Preparation and Metabolite Extraction

For untargeted metabolomic analysis, frozen larvae samples were thawed slowly at 4 °C, and approximately 50–100 mg of each sample was accurately weighed into centrifuge tubes (Biosharp Life Sciences, Hefei, China). Pre-chilled water (200 μL) was added, followed by vortexing for 60 s. Subsequently, 800 μL of pre-chilled methanol (Sigma-Aldrich, St. Louis, MO, USA)/acetonitrile (Sigma-Aldrich, St. Louis, MO, USA) mixture (1:1, *v/v*) was added and samples vortexed again for 60 s. Metabolites were extracted via sonication at low temperature for 30 min and then incubated at −20 °C for 1 h to precipitate proteins and centrifuged at 12,000 rpm for 10 min at 4 °C. The supernatants were collected, vacuum-dried and reconstituted in 200 μL of 30% acetonitrile, vortexed thoroughly and centrifuged at 14,000 rpm for 15 min at 4 °C. The final supernatants were transferred to autosampler vials (Agilent, Santa Clara, CA, USA) for LC–MS/MS analysis. Quality control (QC) samples were prepared by pooling equal aliquots and were used to assess analytical stability [43,44].

#### 2.6.2. LC–MS/MS Analysis

Metabolomic profiling was performed using an ultra-performance liquid chromatography system coupled to high-resolution mass spectrometry (UPLC-HRMS). Chromatographic separations were performed on a reversed-phase Waters HSS-T3 column (100 mm length × 2.1 mm internal diameter, 1.8 μm particle size) maintained at 40 °C. The mobile phase consisted of solvent A (water with 0.1% formic acid) (Merck, Darmstadt, Germany) and solvent B (acetonitrile with 0.1% formic acid), with a flow rate of 0.3 mL min^−1^ and an injection volume of 2 μL. A gradient elution program was applied, starting at 100% A, gradually increasing to 95% B and returning to initial conditions for column re-equilibration. Samples were maintained at 4 °C in the autosampler and analyzed in randomized order, with QC samples injected at regular intervals throughout the run. Mass spectrometric detection was performed using a Q-Exactive HFX high-resolution mass spectrometer (Thermo Scientific, Waltham, MA, USA) equipped with an electrospray ionization (ESI) source operating in both positive- and negative-ion modes. The ion source parameters included a sheath gas flow of 40-arb, auxiliary gas flow of 10-arb, spray voltage of +3000 V (positive mode) or −2800 V (negative mode), capillary temperature of 350 °C and ion-transfer-tube temperature of 320 °C. Data were acquired using Full MS/dd-MS^2^ scan mode over an *m*/*z* range of 70–1050, with a primary mass resolution of 70,000 and a secondary resolution of 17,500.

#### 2.6.3. Metabolomics Data Processing and Analysis

Raw LC-MS/MS data were processed using Progenesis-QI software (v3.0, Nonlinear Dynamics, Newcastle, UK) for baseline correction, peak detection, retention-time alignment and peak normalization. This processing generated a data matrix containing retention time, mass-to-charge ratio (*m*/*z*) and peak intensities. Metabolite identification was conducted by matching accurate mass and MS/MS fragmentation patterns against commercial databases and an in-house metabolite library, with identification confidence assessed based on MS^2^ fragment matching scores. Only metabolites with reliable identification scores were retained for further analysis. Multivariate statistical analyses, including principal component analysis (PCA) and partial least squares discriminant analysis (PLS-DA), were conducted to visualize metabolic differences between temperature treatments. Differential metabolites were identified based on combined statistical significance and multivariate contribution and were subsequently mapped to metabolic pathways to elucidate temperature-induced metabolic reprogramming [43]. Metabolites were annotated based on spectral matching against the reference database, and compounds meeting the predefined similarity threshold were considered identified, whereas those without reliable matches were classified as unidentified.

### 2.7. Transcriptomic Analysis

#### 2.7.1. RNA Extraction and Library Preparation

Total RNA isolated from larvae maintained under three temperature treatments (CT, MCS, SCS) was used for transcriptomic analysis (Wuhan Pronetstest Co., Ltd. Wuhan, China). Frozen larvae were homogenized in TRIzol™ reagent (Invitrogen, Carlsbad, CA, USA) using a high-throughput tissue grinder under RNase-free conditions. Total RNA from *T. absoluta* larvae was extracted using the Animal Tissue/Cell RNA Extraction Kits (N066; Nanjing Jiancheng Bioengineering Institute, Nanjing, China) following the manufacturer’s instructions. RNA purity and concentration were assessed using a NanoDrop spectrophotometer (Nanodrop-2000, Thermo Scientific), while RNA integrity was determined by agarose-gel electrophoresis, and RNA integrity number (RIN) was determined using Agilent-2100 (≥7.0 for library construction). Transcriptomic libraries were constructed using the NEBNext^®^ Ultra™ RNA-Library Prep Kits for Illumina^®^ according to the manufacturer’s protocol. Briefly, polyadenylated mRNA was enriched from total RNA using oligo(dT) magnetic beads. The enriched mRNA was fragmented into short fragments and reverse-transcribed into first-strand cDNA using random hexamer primers, followed by second-strand synthesis to generate double-stranded cDNA. After end repair, A-tailing and adaptor ligation, the cDNA fragments were amplified by PCR, size-selected and quantified to generate sequencing libraries. Each RNA-seq biological sample was prepared from a pool of five larvae, which were stored at −80 °C until RNA extraction.

#### 2.7.2. RNA Sequencing and Quality Control

The prepared libraries were sequenced on an Illumina high-throughput sequencing platform (San Diego, CA, USA) to generate paired-end reads (2 × 150 bp). Raw sequencing reads were subjected to quality filtering using fastp to remove adaptor sequences, low-quality reads, reads containing excessive ambiguous bases and short reads. High-quality clean reads were aligned to the *T. absoluta* reference genome (NCBI GCA_027580185.1, ASM2758018v1); available at: https://www.ncbi.nlm.nih.gov/assembly/GCA_027580185.1 (accessed on 2 January 2026)) using HISAT2 with default parameters. The resulting clean reads were used for downstream analyses. Sequencing quality was evaluated based on standard metrics, including base quality scores, GC content and read length distributions.

#### 2.7.3. Differential Gene Expression and Functional Annotation

Gene-level expression was quantified by counting reads uniquely mapped to annotated exonic regions. Raw read counts were used for differential expression analysis to ensure appropriate statistical modeling. Differentially expressed genes (DEGs) among temperature treatments were identified using DESeq2 based on normalized read counts. Genes with an adjusted *p*-value (Benjamini–Hochberg correction) < 0.05 and |log_2_ fold change| ≥ 1 were considered significantly differentially expressed, and expression levels were normalized as fragments per kilobase of transcript per million mapped reads (FPKM). Functional annotation and enrichment analyses of DEGs were performed using Gene Ontology (GO) and Kyoto Encyclopedia of Genes and Genomes (KEGG) databases. Gene Ontology (GO) enrichment analysis was conducted to identify significantly enriched biological processes, molecular functions and cellular components, while Kyoto Encyclopedia of Genes and Genomes (KEGG) pathway analysis was used to identify metabolic and signaling pathways associated with temperature stress responses. Enrichment significance was determined using adjusted *p*-values < 0.05.

### 2.8. Analysis of Gene Expression Levels

To validate the accuracy and reliability of transcriptomic results, reverse-transcription quantitative PCR (RT-qPCR) was performed on a subset of candidate genes selected from the RNA-Seq analysis, and their primer sequences are provided in Table S1. Selected genes were chosen based on their differential expression and their associations with stress responsive metabolic and adaptive pathways (*n* = 4 tests/treatment). Coding DNA sequences (CDSs) of the target genes were retrieved from the NCBI database (www.ncbi.nlm.nih.gov) based on *T. absoluta* annotation. Gene-specific primers were designed using Primer3-Plus (available at: https://www.primer3plus.com/ (accessed on 12 January 2026)) and primer specificity was first evaluated via in silico BLAST analysis (available at: https://blast.ncbi.nlm.nih.gov/ (accessed on 12 January 2026)) against the *T. absoluta* transcriptome and NCBI databases to exclude potential off-target amplification. Primer specificities were further confirmed by melting curve analysis following RT-qPCR and 2% agarose-gel electrophoresis. Total RNA from *T. absoluta* larvae was extracted using the Animal Tissue/Cell RNA Extraction Kits (N066), as described above, and RNA was reverse-transcribed into complementary DNA (cDNA) using the cDNA first-strand synthesis kit (N118; Nanjing Jiancheng Bioengineering Institute, Nanjing, China). Quantitative PCR was performed using ChamQ™ Universal SYBR qPCR Master Mix (Q711–02, Vazyme Biotech, Nanjing, China) on a Bio-Rad CFX96 RT System (Bio-Rad Laboratories, Hercules, CA, USA). Relative gene expression levels were calculated using the 2^−ΔΔCt^ method, with β-actin serving as the internal reference gene for data normalization (*F*: *CAGGTCCTTACGGATGTC*; *R*: *CTCTTCCAGCCTTCCTTC*) [45,46,47,48]. Gene expression analyses were conducted using four biological replicates per treatment.

### 2.9. Integrative Analysis

For the integrative analysis, pathways significantly enriched in both the transcriptomic and metabolomic datasets were identified, and pathways relevant to cold stress responses were retained. For each shared pathway, we extracted the numbers of DEGs and metabolites contributing to its enrichment. The link values used in the chord diagrams were calculated as the minimum of these two counts, representing the true overlapping strength between the transcriptome and metabolome. Similarly, the integrated network analysis was performed by combining KEGG-enriched transcriptomic and metabolomic datasets. For every comparison, KEGG pathways from genes and metabolites were intersected to retain only pathways shared by both omics layers. Within each shared pathway, KEGG “Input” fields were parsed to extract pathway-assigned genes and their log_2_FC values, while metabolite identifiers were extracted from the metabolomics KEGG enrichment output. The resulting gene–pathway–metabolite relationships were assembled into a tripartite network. Networks were constructed and visualized in Python (v. 3.13.14) using a spring-layout algorithm.

### 2.10. Statistical Analysis

Data were tested for normality using the Shapiro–Wilk test and for homogeneity of variance using Levene’s test before analysis. Differences among treatments were determined using analysis of variance (ANOVA), and treatment means were compared with Tukey’s HSD test (*p ≤* 0.05). All statistical analysis and graphical visualizations were performed using R (version 4.1.0; R Core Team, 2017).

## 3. Results

### 3.1. Low-Temperature Stress Impairs T. absoluta Body Mass and Physiological Performance

**Figure 1:**
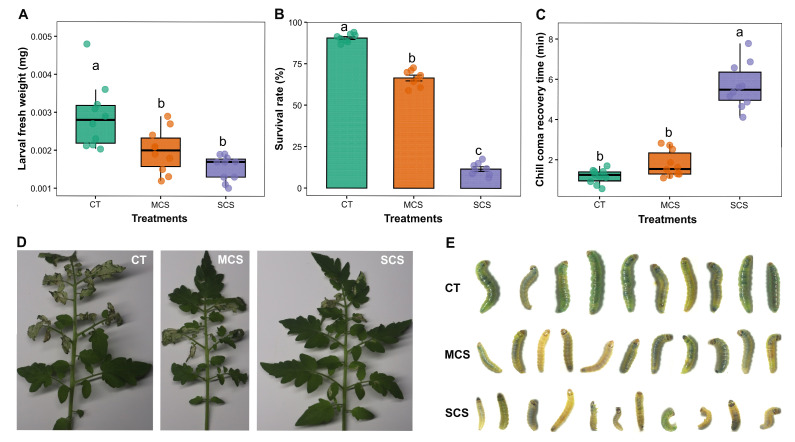
Low-temperature stress exposure influences *T. absoluta* body mass and reduces survival. (**A**) Larval body mass (*n* = 10 replicates/treatment); (**B**) larval survival rates (*n* = 8 replicates/treatment); (**C**) chill-coma recovery time (*n* = 10 replicates/treatment); (**D**) representative leaf photos showing feeding intensity variation across temperature treatments; (**E**) representative larvae photos established across temperature treatments. Data represent mean ± SE and each colored dot represents an individual replicate within each treatment group. Error bars marked with different letters differ significantly among treatments (ANOVA, Tukey’s HSD test, *p* < 0.05).

Low-temperature stress significantly affected *T. absoluta* body mass (*F*_2,27_ = 12.23, *p* = 0.001). SCS-treated larvae showed the most severe reduction in body mass compared to CT (Figure 1A), consistent with the overall stress phenotype. Furthermore, low temperature induced reductions in larval biomass accumulation and decreased feeding performance (*F*_2,27_ = 325.7, *p* = 0.001). The control larvae consumed substantially more host–plant tissue compared to low-temperature-stressed individuals, with MCS reducing consumption and SCS causing severe suppression of feeding behavior (Figure 1D,E and Figure S1). These growth performances were accompanied by reduced larval survival, with MCS reducing survival and SCS causing dramatic mortality (Figure 1B; *F*_2,21_ = 853.2, *p* = 0.001). The physiological impairment induced by low-temperature stress was further evidenced by chill-coma recovery time, where control larvae recovered rapidly from cold exposure, MCS-treated larvae showed extended recovery periods and SCS-exposed larvae displayed substantially prolonged recovery times, indicating progressive cold-induced physiological dysfunction (Figure 1C; *F*_2,27_ = 100.2, *p* = 0.001). These results demonstrate that low-temperature stress operates through multiple integrated mechanistic pathways, simultaneously delaying growth, impairing survival capacities, reducing nutritional acquisition and altering physiological competence, thereby contributing to the compromised phenotype observed in *T. absoluta* larvae under cold stress.

### 3.2. Low-Temperature Stress Alters Larval Biochemical Composition and Digestive Enzyme Activities in T. absoluta Larvae

**Figure 2:**
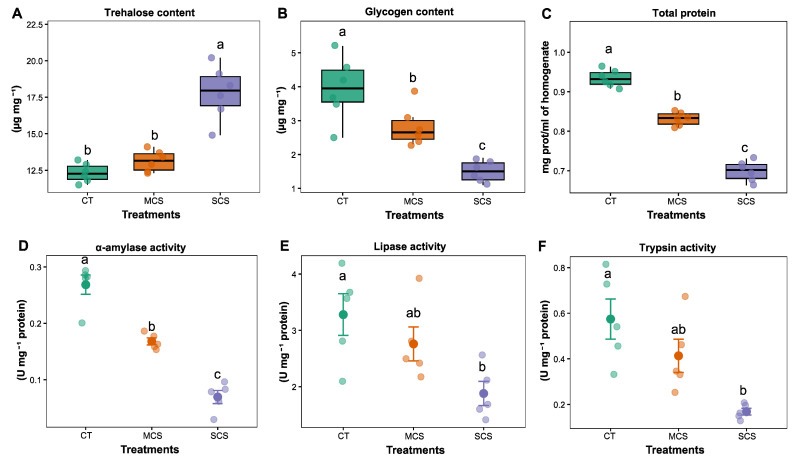
Low-temperature stress exposure induces shifts in metabolic substrates and digestive enzyme activities in third-instar *T. absoluta* larvae. (**A**) Trehalose content; (**B**) glycogen content; (**C**) total protein levels in larvae across temperature treatments (*n* = 6 replicates/treatment); (**D**) α-amylase activity; (**E**) lipase activity; (**F**) trypsin activity (*n* = 5 replicates/treatment). Data represent mean ± SE and each colored dot represents an individual replicate within each treatment group. Different letters above error bars show significant differences among treatments (ANOVA, Tukey’s HSD test, *p* < 0.05).

We examined how low-temperature stress modulates larval metabolic homeostasis and nutritional processing capacities (Figure 2). Low-temperature stress induced marked alterations in larval carbohydrate metabolism (Figure 2A–C). Specifically, trehalose accumulation increased progressively with low-temperature stress intensity (*F*_2,15_ = 35.66, *p* = 0.001), reflecting enhanced cryoprotectant synthesis under cold conditions (Figure 2A). In contrast, glycogen content demonstrated substantial depletion (*F*_2,15_ = 20.23, *p* = 0.001), with MCS-treated larvae showing reduced glycogen stores and SCS-treated larvae displaying severely compromised carbohydrate reserves (Figure 2B). Total protein levels similarly declined under low-temperature stress (*F*_2,15_ = 176.8, *p* = 0.001), with MCS reducing protein abundance and SCS inducing the most dramatic reductions (Figure 2C). These low-temperature-induced metabolic reorganizations were paralleled by significant reductions in digestive enzyme activities (Figure 2D–F). α-amylase activity declined markedly in MCS-treated larvae and was nearly abolished in SCS-exposed individuals (*F*_2,12_ = 64.29, *p* = 0.001), indicating profound suppression of carbohydrate digestion capacity (Figure 2D). Lipase activity decreased moderately under MCS conditions but showed a substantial reduction under SCS exposure (Figure 2E; *F*_2,12_ = 5.495, *p* = 0.020). Trypsin activity similarly decreased with low-temperature stress intensity, with MCS-treated larvae showing reduced proteolytic capacity and SCS-exposed larvae displaying minimal enzyme activities (Figure 2F; *F*_2,12_ = 9.391, *p* = 0.003). These findings reveal that low-temperature stress restructures larval biochemistry through the reallocation of carbohydrate resources toward cryoprotection at the expense of energy storage and systematic suppression of digestive enzyme activities.

### 3.3. Low-Temperature Stress Weakens Antioxidant Defenses and Promotes Oxidative Stress in T. absoluta Larvae

**Figure 3:**
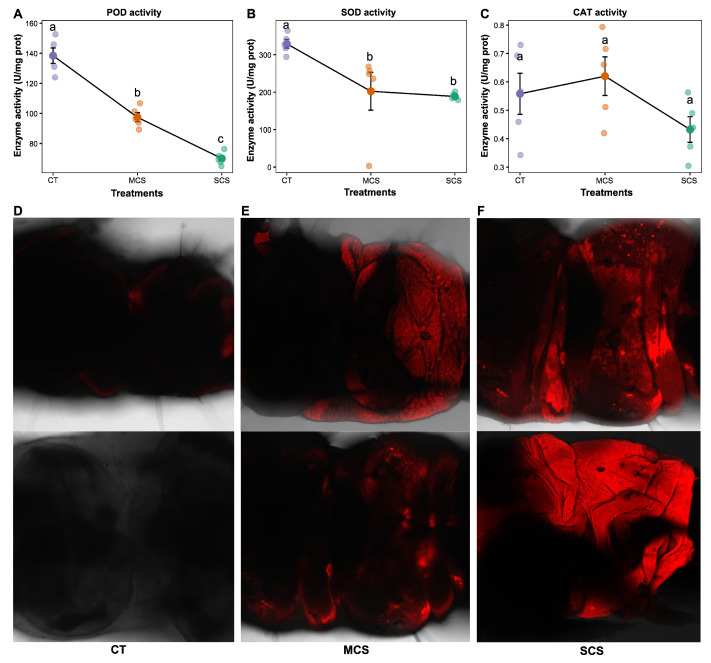
Low-temperature stress exposure increases ROS accumulation and suppresses antioxidant enzyme activities in *T. absoluta* larvae. Third-instar larvae were used for all analyses. (**A**) POD activity; (**B**) SOD activity; (**C**) CAT activity showing variable response across treatment (*n* = 5 replicates/treatment); (**D**–**F**) confocal microscopy imaging of ROS accumulations (red fluorescence) in larval tissues. Data represent mean ± SE and each colored dot represents an individual replicate within each treatment group. Different letters above error bars show significant differences among treatments (ANOVA, Tukey’s HSD test, *p* < 0.05).

Low-temperature stress progressively suppressed the antioxidant defense system of larvae (Figure 3A–C). POD activity declined substantially under MCS and was markedly reduced under SCS (*F*_2,12_ = 89.73, *p* = 0.001), indicating impaired H_2_O_2_ neutralization (Figure 3A). SOD activity was similarly decreased with low-temperature stress, with MCS- and SCS-treated larvae exhibiting reduced activity (*F*_2,12_ = 6.696, *p* = 0.011) (Figure 3B). CAT activity did not differ significantly across treatments, although SCS showed a slight decrease (Figure 3C; *F*_2,12_ = 2.311, *p* = 0.142). These antioxidant enzyme activities were visualized through confocal microscopy. Control larvae showed a minimal ROS signal confined to discrete cellular compartments; MCS treatment exhibited enhanced ROS accumulation in larval tissues, indicating early oxidative stress; while SCS-exposed larvae showed intense and widespread ROS distribution throughout multiple tissue compartments and cellular structures, reflecting severe oxidative stress (Figure 3D–F). The reeducation suggests that thermal stress disrupts the cellular redox balance, potentially triggering oxidative damage and contributing to the developmental and physiological impairments observed under severe cold exposures.

### 3.4. Low-Temperature Stress Induces Extensive Metabolic Reprogramming in T. absoluta Larvae

#### 3.4.1. Metabolomic Profiling and Differential Metabolite Analysis

**Figure 4:**
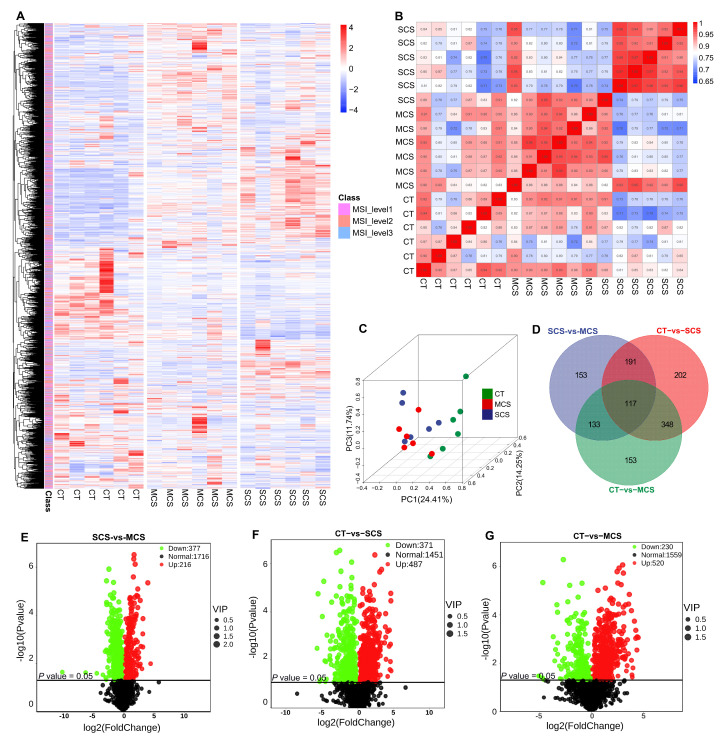
Untargeted metabolomic analysis reveals global metabolic restructuring in *T. absoluta* third-instar larvae. (**A**) Heatmap of normalized metabolite abundances across all samples showing hierarchical clustering and treatment-specific metabolic signature; (**B**) Pearson correlation matrix demonstrating sample clustering patterns and inter-replicate consistency within treatment; (**C**) PCA showing distinct metabolic profiles across treatment; (**D**) Venn diagram displaying the number of significantly altered metabolites; (**E**–**G**) volcano plots displaying significantly upregulated (red) and downregulated (green) metabolites with variable importance in projection scores.

The hierarchical clustering of normalized metabolite abundances showed clear segregation between CT and cold-stressed larvae, with MCS and SCS samples displaying distinct metabolic profiles that progressively diverged from the CT group (Figure 4A). Examination of inter-sample correlations demonstrated that CT samples showed strong positive correlations with each other, indicating consistent and reproducible metabolomic profiles within control larvae. In contrast, MCS and SCS samples displayed progressively weaker correlations both within treatment groups and especially between treatment groups, with SCS samples showing very weak correlation with CT samples, demonstrating that SCS induces metabolomic divergence so profound that SCS larvae develop metabolic profiles essentially orthogonal to the CT condition (Figure 4B). PCA analysis confirmed this pattern, showing clear separation of CT, MCS and SCS clusters in the reduced dimensional space, with SCS samples displaying the greatest separation from control conditions (Figure 4C). Furthermore, there were 153 differential metabolites unique to the SCS-vs-MCS comparison, 202 metabolites unique to CT-vs-SCS and 153 metabolites unique to CT-vs-MCS, indicating treatment-specific metabolic alteration. Notably, 348 metabolites showed alterations in both CT-vs-MCS and CT-vs-SCS comparisons, indicating a dose-dependent metabolic response to low-temperature stress intensities. Also, 117 were common to all three pairwise comparisons, demonstrating that core metabolites are consistently dysregulated across multiple low-temperature stress intensity levels (Figure 4D). Pairwise statistical comparisons identified extensive metabolic dysregulation across all treatments, and the SCS-vs-MCS comparison revealed substantial metabolite alterations, with both increased and decreased metabolites identified (Figure 4E). The CT-vs-SCS comparison showed the most dramatic metabolic divergence, with a large number of differentially abundant metabolites (Figure 4F), reflecting the severe metabolic disruption induced by extreme low-temperature stress. The CT-vs-MCS comparison similarly displayed significant metabolic alteration, though less severe than SCS exposure (Figure 4G). These results indicate that low-temperature stress induces a comprehensive metabolic adjustment in *T. absoluta* larvae that reprograms the key biochemical pathways involved in cold tolerance.

#### 3.4.2. Pathway Enrichment and Alterations in Key Metabolites 

**Figure 5:**
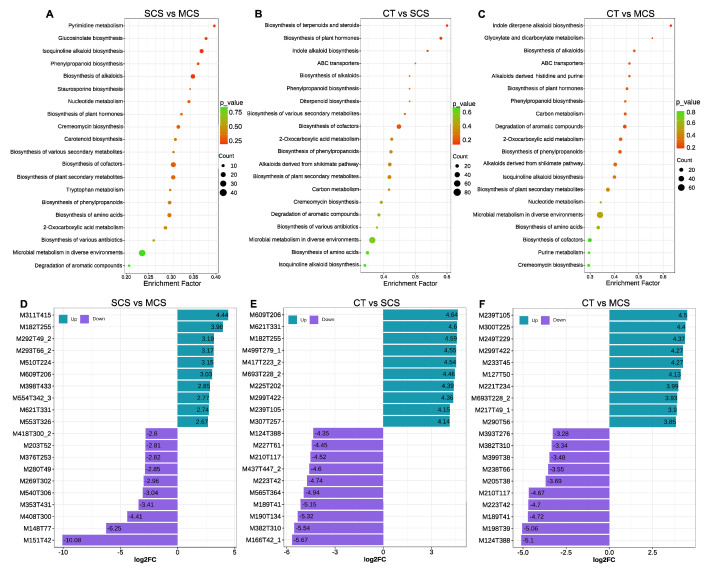
KEGG pathway enrichment analysis of differential metabolites in third-instar *T. absoluta* larvae exposed to low-temperature stress. (**A**–**C**) KEGG plot showing enriched pathways, with bubble size representing the number of detected metabolites and color intensity indicating *p*-value significance; (**D**–**F**) Log_2_ fold-change bar plot of dysregulated metabolites, showing individual metabolite identifier with increased (teal) and decreased (purple) metabolites. Dot size represents the number of differential metabolites associated with each enriched pathway, while dot color indicates the adjusted *p*-value.

Pathway enrichment analysis revealed that low-temperature exposure was associated with alterations in several metabolic pathways related to energy metabolism, biosynthesis and stress-related metabolite changes (Figure 5). In the SCS-vs-MCS comparison, pyrimidine metabolism, glucosinolate biosynthesis, isoquinoline alkaloid biosynthesis, biosynthesis of cofactors, biosynthesis of alkaloids and phenylpropanoid biosynthesis were among the significantly enriched KEGG categories, indicating differential metabolite profiles associated with increasing low-temperature exposure severity (Figure 5A). The CT-vs-SCS comparison identified broader metabolite changes, with differential metabolites assigned to categories, including terpenoid and steroid biosynthesis, plant hormone biosynthesis, indole alkaloid biosynthesis and ABC transporter-related pathways (Figure 5B). The CT-vs-MCS comparison also showed altered metabolite-associated pathways, including indole diterpene alkaloid biosynthesis and alkaloid-related KEGG categories (Figure 5C). These pathway assignments represent database-based metabolite classifications and do not necessarily indicate endogenous biosynthesis of these compounds in *T. absoluta* larvae.

Furthermore, analysis of individual metabolites shows consistent patterns of differential abundance. In the SCS-vs-MCS comparison, metabolites were substantially decreased in SCS larvae, including metabolites annotated to pyrimidine and alkaloid metabolism, indicating reduced abundance of metabolites assigned to these pathways. Conversely, multiple metabolites showed strong increases, predominantly from secondary metabolite pathways (Figure 5D). In the CT-vs-SCS comparison, decreased metabolites were predominantly from primary metabolic pathways and biosynthetic routes, indicating specific secondary metabolite increases (Figure 5E). The CT-vs-MCS comparison showed more moderate but consistent dysregulation patterns, confirming the stress-dependent nature of metabolic responses (Figure 5F).

Furthermore, the metabolomic dataset comprised diverse chemical classes reflecting the comprehensive nature of the metabolite composition (Figure S2). Lipids and lipid-like molecules constituted the largest superclass, followed by organic acids and derivatives and organoheterocyclic compounds, indicating altered abundance patterns of metabolites associated with secondary metabolism. Detailed subclass analysis revealed that amino acids, carbohydrates and fatty acids formed major components, alongside specialized classes, including carboxylic acids and derivatives, prenol lipids, fatty acyls and organooxygen compounds, which showed stress-dependent fluctuations (Figure S2A,B). Dynamic trajectory analysis of metabolite subclasses across treatments revealed divergent abundance patterns: Subclass 5 showed progressive suppression from the SCS to CT condition, whereas Subclass 6 and Subclass 7 showed progressive upregulation with increasing low-temperature stress (Figure S3). These opposing trajectories indicate coordinated suppression of energy-intensive metabolic processes coupled with increased abundance of metabolites potentially associated with stress responses.

### 3.5. Low-Temperature Stress Triggers Coordinated Transcriptional Reprogramming in T. absoluta Larvae

#### 3.5.1. Global Gene Expression Profiling and Differential Expression Analysis

**Figure 6:**
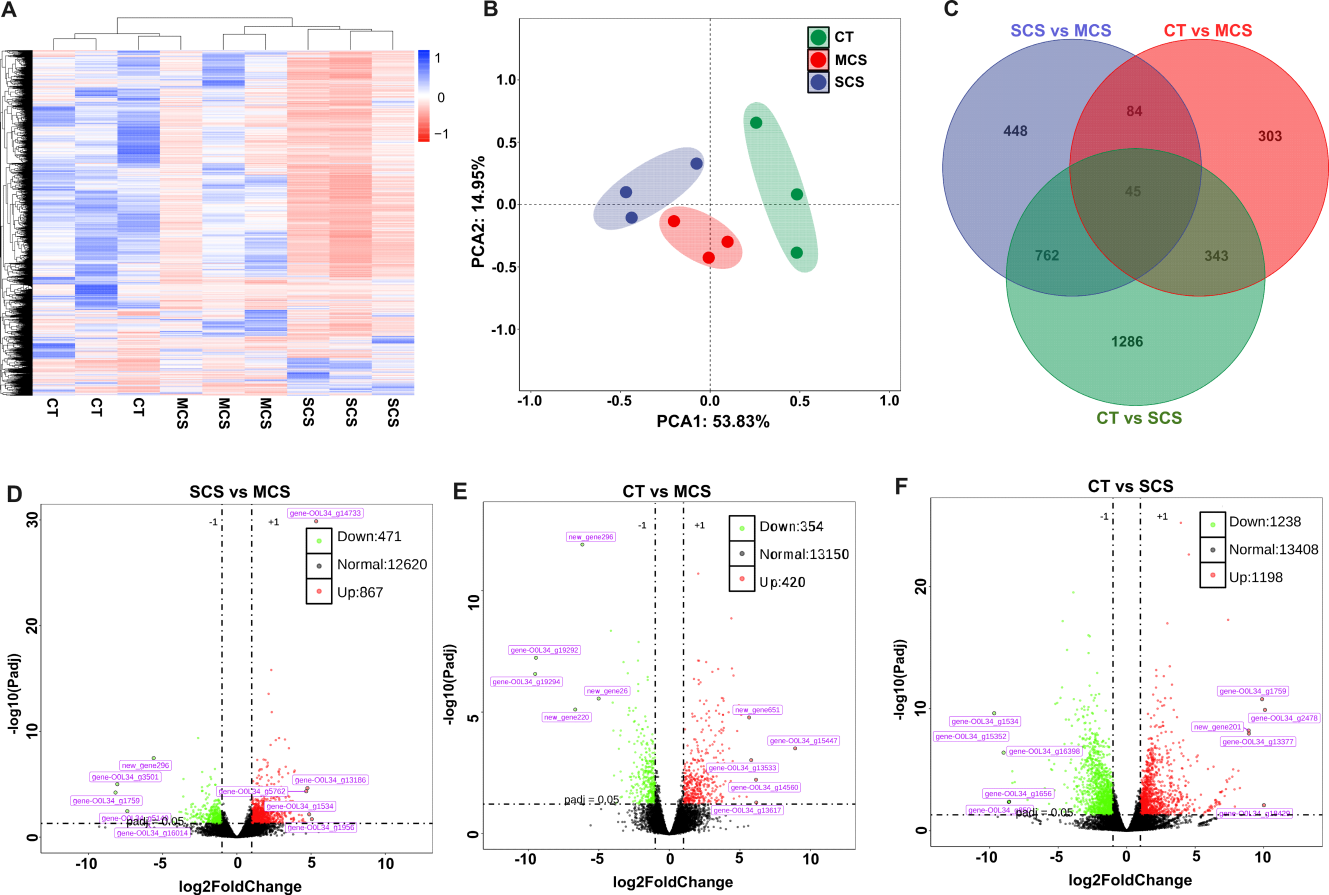
Transcriptomic profiling reveals extensive gene expression restructuring in third-instar *T. absoluta* larvae under low-temperature stress. (**A**) Heatmap of normalized gene expression level across all samples showing hierarchical clustering and treatment-specific transcriptional signature; (**B**) PCA demonstrating distinct transcriptomic profiles across treatment; (**C**) Venn diagram showing the number of significantly altered genes in pairwise comparison; (**D**–**F**) volcano plot showing significantly downregulated (green) and upregulated (red) transcripts.

Hierarchical clustering of normalized gene expression levels showed clear segregation between control and cold-stressed larvae, with MCS and SCS samples displaying distinct transcriptomic profiles that progressively diverged from the control phenotype (Figure 6A). Also, inter-sample correlation analysis showed strong within-treatment clustering and substantially reduced correlation coefficients between different treatments (Figure S4A), indicating temperature-dependent transcriptional reorganizations. PCA analysis confirmed substantial transcriptional divergence, showing clear separation of CT, MCS and SCS clusters in the reduced dimensional space, with SCS samples displaying the greatest separation from CT (Figure 6B). Venn diagram analysis identified 448 DEGs unique to the SCS-vs-MCS comparison, 303 unique to CT-vs-MCS and 1286 unique to CT-vs-SCS (Figure 6C). Notably, 343 genes showed dysregulation in both the CT-vs-MCS and CT-vs-SCS comparison. The presence of 84 genes altered in both the SCS-vs-MCS and CT-vs-MCS comparison and 762 genes dysregulated in both the SCS-vs-MCS and CT-vs-SCS comparison further highlights the transcriptional response with hierarchical activation of stress response pathways.

Pairwise statistical comparisons identified extensive transcriptional dysregulation across all treatment contrasts. The SCS-vs-MCS comparison revealed substantial gene expression alterations, with 471 downregulated and 867 upregulated genes identified, indicating progressive transcriptional reorientation with increasing low-temperature stress severity (Figure 6D). The CT-vs-MCS comparison showed moderate gene expression changes, with 354 downregulated and 420 upregulated genes, reflecting the intermediate physiological stress phenotype (Figure 6E). The CT-vs-SCS comparison displayed the most dramatic transcriptional divergence, with 1238 downregulated and 1198 upregulated genes, demonstrating the greatest transcriptional divergence under extreme low-temperature stress (Figure 6F).

#### 3.5.2. Functional Enrichment and Transcriptional Reprogramming

**Figure 7:**
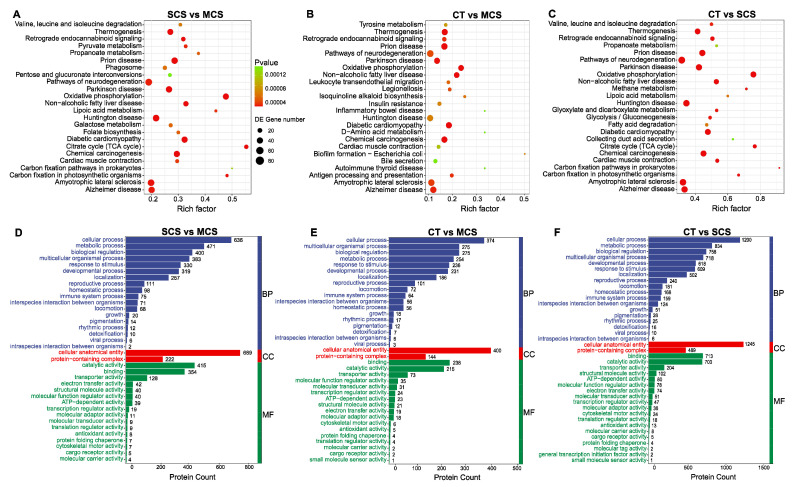
Functional enrichment of genes differentially expressed in third-instar *T. absoluta* larvae under low-temperature stress. (**A**–**C**) KEGG enrichment showing enriched pathways, with bubble size representing gene count and color intensity indicating *p*-value significance; (**D**–**F**) gene ontology analysis showing biological processes (blue), cellular components (red) and molecular functions (green) with corresponding protein counts. Dot size represents the number of differentially expressed genes associated with each KEGG pathway, whereas dot color indicates the enrichment significance (adjusted *p*-value).

Low-temperature stress altered several metabolic and stress response pathways associated with larval homeostasis (Figure 7). In the SCS-vs-MCS comparison, thermogenesis, neurodegeneration, oxidative phosphorylation and amyotrophic lateral sclerosis signaling emerged as significantly enriched pathways, indicating metabolic reorientations toward amino acid catabolism with increasing cold stress severity (Figure 7A). In the CT-vs-MCS comparison, thermogenesis, oxidative phosphorylation, prion disease, amino acid metabolism and Alzheimer’s disease signaling were among the most significantly affected pathways (Figure 7B). The CT-vs-SCS comparison revealed the most extensive pathway dysregulation, with valine, leucine and isoleucine degradation, thermogenesis, citrate cycle and prion disease pathways showing dramatic enrichment (Figure 7C). Gene ontology analysis further characterized the functional scope of low-temperature stress inducing transcriptional changes. In the SCS-vs-MCS comparison, cellular processes and metabolic processing dominated the biological process category, with developmental processes and multicellular organismal processes prominently altered. Cellular component analysis showed cellular anatomical entity and protein-containing complex as major targets. Molecular function categories highlighted catalytic activity and binding functions (Figure 7D).

In the CT-vs-MCS comparison, multicellular organismal process, cellular process and biological regulation dominated the biological process category, while cellular anatomical entity and protein-containing complex were the primary cellular component targets and binding and catalytic activity the most extensively altered molecular functions, indicating diverse transcriptional responses spanning developmental and metabolic processes (Figure 7E). The CT-vs-SCS comparison exhibited the most extensive GO enrichment across all three biological dimensions, with cellular process, metabolic processes and biological regulation showing the largest protein counts, cellular anatomical entity and protein-containing complexes as predominant cellular component targets and binding and catalytic activity as the most dominantly altered molecular functions, reflecting comprehensive transcriptional restructuring, affecting essentially all major biological systems under severe low-temperature stress (Figure 7F). Transcription factor analysis identified several predicted regulatory factors associated with the differentially expressed genes. The ten highest-ranked factors were *SMARCA1*, *CENPA*, *DNAJC1*, *BAZ2B*, *ZNFX1*, *ZNF516*, *BCL11B*, *NSD2*, *L3MBTL3* and *HESI* (Figure S4B). Also, the comprehensive pathway analysis revealed that SCS exposure primarily affects cellular processes, signal transduction and metabolic pathways, with similar pathway categories enriched in CT-vs-MCS and CT-vs-SCS comparisons but at progressively increasing scales (Figure S5A–C). The regulatory network analysis demonstrated complex associations among gene families, predicted transcription factors and target genes, with substantial rewiring between treatments: the SCS-vs-MCS comparison showed distinct regulatory architecture, the CT-vs-MCS comparison revealed intermediat network complexity and the CT-vs-SCS comparisons exhibited the most extensive network reorganization, with diverse gene families and transcription factors involved in the stress response (Figure S6A–C). These transcriptomic analyses demonstrate that low-temperature stress triggers systematic gene expression reprogramming with progressive intensification of transcriptional dysregulation as temperature decreases.

### 3.6. Integrative Metabolomic–Transcriptomic Analysis Reveals Key Metabolic and Gene Expression Networks

**Figure 8:**
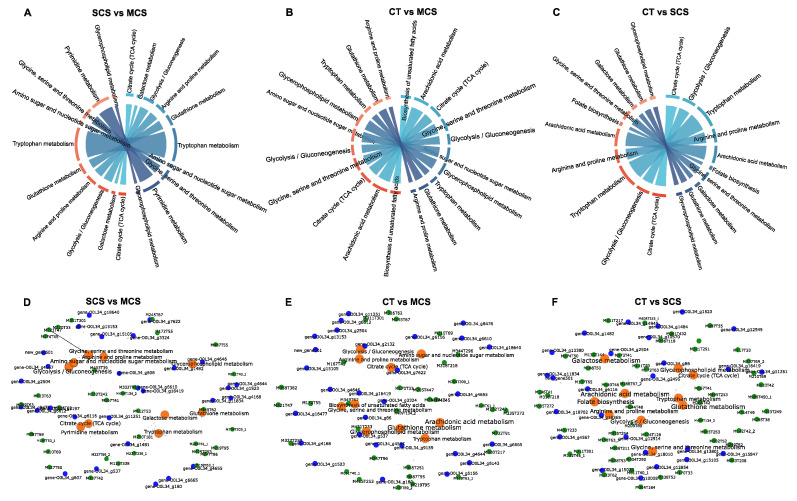
Low-temperature stress in third-instar *T. absoluta* larvae. (**A**–**C**) Chord diagram showing metabolic pathway association. Blue arcs represent genes, while orange arcs represent metabolite pathways, with chord thickness reflecting the number of gene–metabolite–pathway connections; (**D**–**F**) gene–metabolite interaction network showing individual genes (blue), metabolites (green) and KEGG pathways (orange), with edges representing functional associations.

We integrated the differential metabolite and gene expression datasets and mapped their functional relationships to shared KEGG pathways (Figure 8). In the SCS-vs-MCS comparison, the chord diagram displayed moderate connections among genes, metabolites and pathways, with amino sugar metabolism (tryptophan metabolism, arginine and proline metabolism), carbohydrate metabolism (glycolysis/gluconeogenesis) and nucleotide sugar metabolism emerging as principal pathways. The thickness of chords connecting metabolites to these pathways indicated that multiple dysregulated metabolites map to each core pathway, demonstrating multiple altered features in these fundamental metabolic pathways (Figure 8A). The CT-vs-MCS comparison revealed intermediate pathway network complexity, with similar core metabolic pathways showing enrichment alongside glycerophospholipid metabolism and arachidonic acid metabolism pathways (Figure 8B). The CT-vs-SCS comparison exhibited the most extensive network density, with dramatically increased chord density, indicating that SCS generates dysregulation across a much larger metabolite pathway association network. The expanded metabolite pathway connections in this comparison reflected comprehensive remodeling of amino sugar metabolism, carbohydrate metabolisms, nucleotide metabolisms and arachidonic acid metabolisms, demonstrating that extreme cold stress triggers wholesale metabolic pathway engagement rather than isolated metabolite perturbation (Figure 8C).

Furthermore, the gene–metabolite interaction networks provided a more detailed representation of the transcriptional metabolic coordination underlying this pathway-level association. In the SCS-vs-MCS comparison, genes and metabolites clustered within functional modules corresponding to specific metabolic pathways. Genes encoding amino sugar catabolic enzymes clustered with their corresponding metabolite products, demonstrating transcriptional–metabolic coupling: upregulation of catabolic enzyme genes directly produces the dysregulated amino acid-derived metabolites (Figure 8D). The CT-vs-MCS comparison network displayed intermediate network connectivity with emerging stress responsive metabolic modules: genes encoding glycolysis enzymes began to cluster distinctly from developmental and biosynthetic genes, reflecting the initiation of metabolic reorientation under moderate stress (Figure 8E). The CT-vs-SCS comparison network exhibited maximal complexity, with extensive hub formation at the pathway intersection. Highly connected hub genes appeared at intersections of multiple metabolic pathways (folate metabolism, galactose metabolism), indicating these genes serve as regulatory integration points coordinating metabolic flux across multiple pathways (Figure 8F). These integrative metabolomic–transcriptomic networks demonstrate that low-temperature stress orchestrates metabolite accumulation and gene expression changes that are systematically organized around core metabolic pathways. Also, the identification of individual key metabolites and genes indicates low-temperature altered metabolites involved in central carbon metabolism, amino acid turnover, lipid remodeling and membrane-related processes. Consistently, several differentially expressed genes associated with mitochondrial energy production, oxidation–reduction reactions, ion balance, cytoskeletal organization and cellular signaling were strongly regulated. These results indicate that low temperature disrupts energy supply while also affecting membrane stability and cellular functions (Table 1).

### 3.7. Low-Temperature Stress Alters Candidate Gene Expression and Proline Content in T. absoluta Larvae

**Figure 9:**
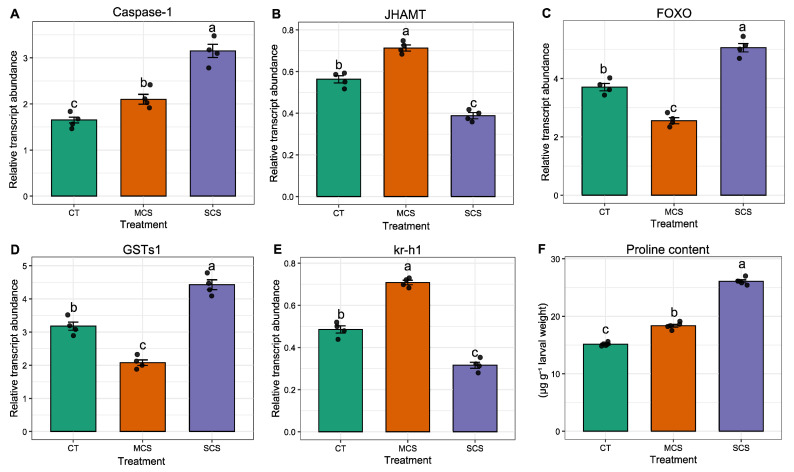
Candidate gene expression and biochemical quantification of proline in third-instar *T. absoluta* larvae under low-temperature stress. (**A**) Relative transcript abundance of Caspase-1; (**B**) JHAMT; (**C**) FOXO; (**D**) GSTs1; (**E**) kr-h1 (*n* = 4 replicates/treatment); (**F**) larval proline content (*n* = 5 replicates/treatment). Error bars marked with different letters differ significantly among treatments and each dot represents an individual replicate within each treatment group (ANOVA, Tukey’s HSD test, *p* < 0.05).

We performed metabolite quantification and RT-PCR of selected candidate genes identified as central regulatory nodes in the low-temperature stress response (Figure 9). Caspase-1 showed progressive upregulation across increasing cold stress intensity, with minimal transcript abundance in CT larvae and maximal expression under SCS (Figure 9A; *F*_2,9_ = 48.48, *p* = 0.001). JHAMT displayed elevated expression under MCS with substantial suppression under SCS (Figure 9B; *F*_2,9_ = 105.5, *p* = 0.001). FOXO showed marked dose-dependent upregulation, with maximum expression under SCS conditions (Figure 9C; *F*_2,9_ = 97.19, *p* = 0.001). GSTs1 expression decreased under MCS and increased under SCS (Figure 9D; (*F*_2,9_ = 91.74, *p* = 0.001)). kr-h1 showed a similar pattern to JHAMT, with elevated expression under MCS and suppression under SCS (Figure 9E; *F*_2,9_ = 186.6, *p* = 0.001). These results confirmed the transcriptomic predictions, validating that the gene expression changes detected through RNA-sequencing translate into measurable changes in transcript abundance detectable by independent quantitative approaches. Furthermore, independent biochemical quantification of larval proline content confirmed the metabolomics prediction of low-temperature stress-induced proline accumulation (Figure 9F). Control larvae maintained normal proline levels, whereas MCS-treated larvae showed intermediate accumulation and SCS-exposed larvae displayed maximal proline contents (*F*_2,12_ = 604.1, *p* = 0.001). This progressive temperature-dependent increase validates the metabolomic findings, showing elevated proline abundance under low-temperature stress.

## 4. Discussion

The tomato leafminer, *T. absoluta*, one of the most economically damaging lepidopteran pests worldwide, has demonstrated remarkable adaptability to diverse climatic regions, yet the comprehensive molecular mechanisms supporting its cold tolerances remain poorly characterized [49,50]. Low-temperature stress presents a fundamental physiological challenge to ectothermic insects that extends far beyond simple metabolic slowing, and these organisms have evolved multilayered defense mechanisms to cope with such complexities [51].

Our results showed that prolonged low-temperature exposure progressively reduced final larval fresh body mass, survival and feeding performance, with the strongest effects observed under SCS. CCRT was also prolonged under low-temperature exposure, indicating impaired physiological performance and reduced tolerance to thermal challenge. These observations show that low-temperature stress imposes fundamental constraints on larval physiology, causing physiological adjustments associated with reduced metabolic activity and resource reallocation under prolonged low-temperature exposure [52]. Under restrictive conditions, larvae reallocate resources from growth toward essential maintenance processes, yet the concurrent decline in survival suggests that cold exposure surpasses their physiological limits, revealing a threshold beyond which normal development cannot be sustained [6,9,53]. Because endpoint analyses were conducted on surviving larvae, particularly under the 5 °C treatment, the observed molecular profiles may partly reflect survivor selection. The concurrent reductions in feeding and digestive enzyme activities provide a plausible mechanistic explanation for impaired larval growth under cold stress. The activities of digestive enzymes such as α-amylase, lipase and trypsin were progressively suppressed following low-temperature exposure. These enzymes are essential for nutrient acquisition from the host plant; their reduced activities directly impair the capacity of the larvae to process and absorb nutrients [54]. Simultaneously, feeding behaviors were suppressed, as evidenced by the reduced leaf area consumed under low-temperature stress. By simultaneously reducing feeding, digestion and energy-demanding processes, low-temperature stress creates a metabolic bottleneck and may reduce the anabolic activity of anabolic metabolism, ultimately forcing larvae to reallocate resources away from growth [55,56,57]. Although the present study focused on larval responses, temperature-induced changes in host plant physiology and leaf quality may also contribute to altered feeding behavior and should be further investigated through integrated plant–insect analyses.

These metabolic reorientations are reflected in dramatic shifts in biochemical composition under low-temperature stress. Glycogen, the primary carbohydrate energy storage compound [58], was severely depleted under low temperature, indicating the mobilization of stored energy reserves. However, the depleted glycogen was not simply consumed for immediate energy production. Instead, biochemical analysis revealed trehalose accumulation, a disaccharide with known protective properties that stabilize cellular structures at low temperature [59,60]. Simultaneously, total protein content declined, while specific amino acid-derived metabolites accumulated, indicating that protein was similarly redirected toward protective rather than energy-generating purposes. This metabolic reallocation, from energy storage toward biosynthesis of protective compounds, indicates that low temperature triggers fundamental reorganization of metabolic priorities. Rather than expending stored energy to fuel development and growth, larvae may divert these resources toward synthesis of compounds that enhance cellular protection [61]. Furthermore, our results showed that larval antioxidant enzymes including POD, SOD and CAT were progressively altered under low-temperature stress. Notably, these altered enzyme activities correlated with marked accumulation of ROS in larval tissues, with SCS producing intense and widespread ROS in the larval bodies. Oxidative stress can arise through multiple mechanisms: reduced enzymatic activities at lower temperatures may impair antioxidant defense capacities, metabolic shifts toward catabolic pathways may generate increased ROS through altered mitochondrial functions and direct cellular injury from cold exposures may trigger additional ROS generation [19]. This suggests that larval cold tolerance is limited not only by metabolic constraints but also by its ability to withstand oxidative stress, an observation with important implications for understanding how environmental stresses limit pest population persistence [62,63].

Furthermore, *T. absoluta* larval metabolomic profiling revealed that temperature-regulated physiological changes and biochemical alterations in larval physiology are supported by changes in metabolite abundances, which helps insects cope with low-temperature stress through metabolic restructuring [17]. All the treatment groups displayed differentially induced metabolites across the temperature gradients, with distinct metabolomic signatures that varied according to stress severity. Control larvae maintained consistent metabolomic profiles with strong inter-replicate correlation, whereas MCS and SCS larvae showed progressively weaker metabolomic correlation, both within and between treatments, indicating that SCS induces metabolomic divergence so profound that stressed larvae develop metabolic profiles fundamentally different from CT [64]. Also, metabolites showed coordinated increases and decreases when compared with CT, with systematic patterns of upregulation and downregulation reflecting organized metabolic restructuring rather than nonspecific metabolite perturbation. These results suggest that dysregulated metabolites may play critical roles in larval thermal adaptations under low temperatures, potentially functioning as cryoprotectants, osmolytes and signaling molecules that enable cellular survival under thermal challenges [65,66]. Furthermore, KEGG pathway enrichment analysis of these dysregulated metabolites revealed significant associations with several metabolic categories, including glucosinolate-related pathways, pyrimidine metabolism and alkaloid-related pathways, under low-temperature exposure. These metabolite-associated pathway categories suggest that low-temperature exposure alters how larvae process and utilize metabolic substrates; however, their biological functions require cautious interpretation because pathway assignments are based on database annotations [17,67,68]. The enrichment of alkaloid- and glucosinolate-related KEGG categories should be interpreted cautiously, as these annotations may reflect diet-derived metabolites, conserved metabolic transformations or database-derived pathway assignments rather than confirmed endogenous biosynthetic activity in *T. absoluta* larvae.

Moreover, the observed physiological and biochemical alterations align with a broader system-level reprogramming of gene expression, as demonstrated by our transcriptomic analysis [49]. Gene expression profiles differed among the treatment groups, with genes showing differential expression across the temperature gradient. Under SCS, higher numbers of upregulated and downregulated genes were observed compared to MCS or CT, suggesting that low-temperature stress progressively engages more extensive transcriptional remodeling as temperature decreases. Also, control larvae maintained consistent genes expression profiles, while low-temperature stress larvae showed progressively divergent transcriptomic signatures, and all the genes were uniquely dysregulated at different stress intensities. These patterns indicate that low-temperature stress activates both core genes consistently affected across all stress levels and stress-intensity-specific genes preferentially engaged under severe thermal challenges [22,49,69]. Furthermore, the pathway enrichment analysis revealed specific metabolic and signaling pathways whose dysregulated genes indicate system-level responses to low-temperature stress. Pathways, including thermogenesis signaling, retrograde endocannabinoid signaling, amino acid metabolism pathways and prion disease pathways, showed significant enrichment across the treatments. These diverse enriched pathways suggest that dysregulated genes are not randomly distributed but rather cluster in functionally coherent routes important for cellular survival under thermal stresses [69]. These multi-level dysregulations of genes across biological processes, cellular components and molecular functions suggest that low-temperature stress operates as a comprehensive system-level stressor affecting cellular organization and function at multiple biological scales [70,71,72].

The integrative metabolomic–transcriptomic analysis revealed coordinated associations between transcriptional changes and metabolic reorientation, suggesting that altered gene expression patterns are linked with changes in metabolite abundance under low-temperature stress [22,68]. The metabolite–pathway–gene network showed that dysregulated genes were enriched within metabolic pathways, with several metabolic genes associated with corresponding metabolite alterations [24,73,74]. Furthermore, RT-PCR analysis of specific genes related to *T. absoluta* growth and development confirmed that transcriptomic predictions accurately reflected changes in transcript abundances. For instance, the gene caspase-1, which plays an important role in *T. absoluta* larval growth and development, was highly expressed in SCS, indicating a shift toward enhanced apoptotic or stress-associated cellular turnover under low-temperature stress [46]. The biochemical quantification of proline confirmed the metabolically predicted increases in proline abundance as measurable changes in larval physiology [75]. The consistency between multi-omics patterns and independent physiological measurements supports the reliability of the identified molecular and metabolic responses under low-temperature stress.

## 5. Conclusions

In summary, third-instar *T. absoluta* larvae exhibited coordinated physiological, metabolic and transcriptional responses to prolonged low-temperature exposure. Cold stress reduced feeding body mass, survival and physiological performance; suppressed digestive enzyme activities; depleted glycogen and protein reserves; and increased the accumulation of trehalose, proline and reactive oxygen species. Integrative multi-omics analysis indicated coordinated changes in gene expression and metabolite abundance, consistent with a shift in resource allocation from growth-related processes toward cellular maintenance and stress protection. These findings advance our understanding of the physiological and molecular basis of cold sensitivity in *T. absoluta* and provide a foundation for future studies investigating how low-temperature responses influence the seasonal persistence and geographic establishment of this invasive pest under changing climatic conditions.

## Figures and Tables

**Table 1:** Response patterns of selected metabolites and genes associated with energy metabolism, amino acid metabolism, membrane processes and cuticle or cytoskeletal structure in larvae under low-temperature stress.

Category	Feature	Class	CT vs. MCS	CT vs. SCS	SCS vs. MCS	Annotation/Function
Carbohydrate and central energy metabolism	2-Phosphoglyceric acid	Metabolite	↓ −3.00	↓ −0.58	↓ −2.42	Central carbon metabolism and energy supply
	6-Phosphonoglucono-D-lactone	Metabolite	↑ 2.30	↑ 2.66	↓ −0.36	Central carbon metabolism and energy supply
	Citric acid	Metabolite	↓ −3.82	↓ −3.27	↓ −0.55	Central carbon metabolism and energy supply
	Neohesperidose	Metabolite	↑ 4.14	↑ 3.06	↑ 1.08	Central carbon metabolism and energy supply
Energy metabolism and oxidation-reduction	gene-O0L34_g13046 (ATP synthase-coupling factor 6, mitochondrial)	Gene	↑ 1.12	↑ 3.00	↑ 1.85	ATP synthase-coupling factor 6
	gene-O0L34_g15029 (Glucose dehydrogenase)	Gene	↑ 2.13	↑ 4.72	↑ 2.57	Glucose dehydrogenase
	gene-O0L34_g15038 (NADH dehydrogenase)	Gene	↑ 1.09	↑ 2.29	↑ 1.18	NADH dehydrogenase
	gene-O0L34_g1545 (Cytochrome b-c1 complex subunit Rieske, mitochondrial)	Gene	↑ 1.16	↑ 2.31	↑ 1.13	Cytochrome b-c1 complex subunit Rieske
	gene-O0L34_g15531 (NADH dehydrogenase)	Gene	↑ 1.22	↑ 2.49	↑ 1.24	NADH dehydrogenase
	gene-O0L34_g16254 (NADH dehydrogenase)	Gene	↑ 1.09	↑ 2.29	↑ 1.18	NADH dehydrogenase
	gene-O0L34_g6116 (Enolase)	Gene	↑ 1.85	↑ 3.28	↑ 1.41	Enolase; Domain: Enolase, C-terminal TIM barrel domain
	gene-O0L34_g8983 (NADH dehydrogenase)	Gene	↑ 1.28	↑ 2.36	↑ 1.06	NADH dehydrogenase
Amino acid and peptide metabolism	1H-Indole-2,3-dione	Metabolite	↓ −2.30	↓ −1.48	↓ −0.82	Amino acid/indole metabolism
	2-(5-Hydroxy-1H-indol-2-yl)acetic acid	Metabolite	↓ −2.30	↓ −1.48	↓ −0.82	Peptide/amino acid turnover and nitrogen redistribution.
	3,4-Dihydroxymandelaldehyde	Metabolite	↑ 2.01	↑ 2.86	↓ −0.85	Peptide/amino acid turnover and nitrogen redistribution
	Glutamyl tryptophan	Metabolite	↓ −2.62	↓ −1.19	↓ −1.43	Amino acid/indole metabolism
Lipid and membrane-related metabolism	2-Arachidonyl Glycerol ether	Metabolite	↓ −5.54	↓ −3.34	↓ −2.19	Lipid remodeling
	8-Amino-7-oxononanoic acid	Metabolite	↓ −2.25	↓ −0.76	↓ −1.49	Lipid remodeling
Membrane transport and ion balance	gene-O0L34_g14496 (Actin, muscle-type A2)	Gene	↑ 1.27	↑ 3.67	↑ 2.40	Actin, muscle-type A2
	gene-O0L34_g7907 (Trimeric intracellular cation channel type 1B.1)	Gene	↑ 1.98	↑ 4.00	↑ 2.00	Trimeric intracellular cation channel type 1B.1
Structure, cuticle and cytoskeleton	gene-O0L34_g13794 (Alpha-actinin, sarcomeric)	Gene	↑ 1.49	↑ 3.03	↑ 1.51	Alpha-actinin, sarcomeric
Signal transduction and gene regulation	gene-O0L34_g10052 (Mitogen-activated protein kinase 15)	Gene	↑ 1.27	↑ 2.39	↑ 1.13	Mitogen-activated protein kinase 15
	gene-O0L34_g2604 (Insulin-like growth factor 2 mRNA-binding protein 2)	Gene	↑ 2.32	↑ 3.95	↑ 1.65	Insulin-like growth factor 2 mRNA-binding protein 2
Cofactor and cellular stress-related metabolism	p-Aminobenzoic acid	Metabolite	↓ −1.80	↓ −1.06	↓ −0.74	Supporting cellular stress response
Oxidation-related and aromatic metabolites	4-Nitrophenol	Metabolite	↓ −1.89	↓ −1.24	↓ −0.66	Aromatic/oxidation-related metabolite
Nucleotide metabolism	Allantoic acid	Metabolite	↑ 2.13	↑ 3.46	↓ −1.32	Cellular repair, or growth-related metabolism

Arrows indicate the direction of change relative to the first condition listed in each comparison (↑, increased abundance or expression; ↓, decreased abundance or expression). Numerical values represent log_2_ fold changes.

## Data Availability

The original contributions presented in this study are included in the article and Supplementary Materials. No additional datasets were generated or analyzed beyond those presented in the manuscript, Supplementary Materials and submitted supporting files. Further inquiries can be directed to the corresponding author.

## Supplementary Materials

The following supporting information can be downloaded at: https://www.mdpi.com/article/10.3390/biology15151308/s1, Figure S1. Leaf area mined by *T. absoluta* larvae under low-temperature stress. Data represent mean ± SE. Error bars marked with different letters differ significantly among treatments (ANOVA, Tukeys-HSD test, *p* < 0.05); Figure S2. Comprehensive classification of detected metabolites by chemical structure and metabolic origin. (A) Distribution of metabolites by major class; (B) Subclass categorization; (C) Super-class distribution. “N/A” indicates metabolites detected in the dataset for which chemical superclass or subclass information was unavailable based on the applied annotation database; Figure S3. Dynamic expression patterns of metabolite subclasses across temperature treatments. Each subplot represents a distinct metabolite subclass with individual metabolites shown as separate lines and the bold black line indicating the mean trajectory; Figure S4. Transcription factor analysis and sample correlation. (A) Pearson correlation matrix demonstrating high inter-replicate consistency within treatments; (B) Pie-chart showing the distribution of differentially expressed genes annotated to the top 10 transcription factor families; Figure S5. (A–C) Comprehensive KEGG pathway enrichment analysis highlighting cellular processes, environmental information processing, genetic information processing, metabolism, human diseases and organismal systems categories across all treatment comparisons; Figure S6. Gene family–transcription factor–gene regulatory networks. (A–C) Sankey diagrams illustrating the complex regulatory networks connecting gene families), transcription factors and individual genes across treatments comparisons; Table S1. Forward (*F*) and reverse (*R*) primer sequences used in real-time quantitative PCR.

## Abbreviations

The following abbreviations are used in this manuscript:

CT	Control stress
MCS	Moderate cold stress
SCS	Severe cold stress
DEGs	Differentially expressed genes
