# Supplementary material for: Elucidating Cold-Stress-Induced Metabolic and Transcriptional Reprogramming in Tuta absoluta Larvae Through Integrated Multi-Omics Analysis
Source: Biology (Basel). 2026 Aug 5;15(15):1308. doi: 10.3390/biology15151308 (PMC13463870; doi:10.3390/biology15151308)
Supplement: Supplementary file 1 [file biology-15-01308-s001.zip › biology-4448203-supplementary.pdf]

Supporting Information

Elucidating Cold-Stress-Induced Metabolic and Transcription-al Reprogramming in *Tuta absoluta* Larvae Through Integrated Multi-Omics Analysis

The following Supporting Information is available for this article:

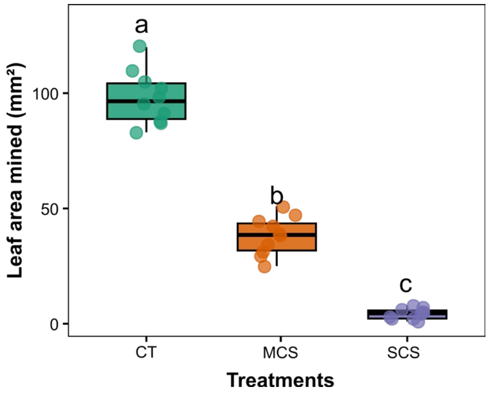

Fig. S1. Leaf area mined by *T. absoluta* larvae under low temperature stress. Data represent mean ± SE. Error bars marked with different letters differ significantly among treatments (ANOVA, Tukeys-HSD test, $p < 0.05$).

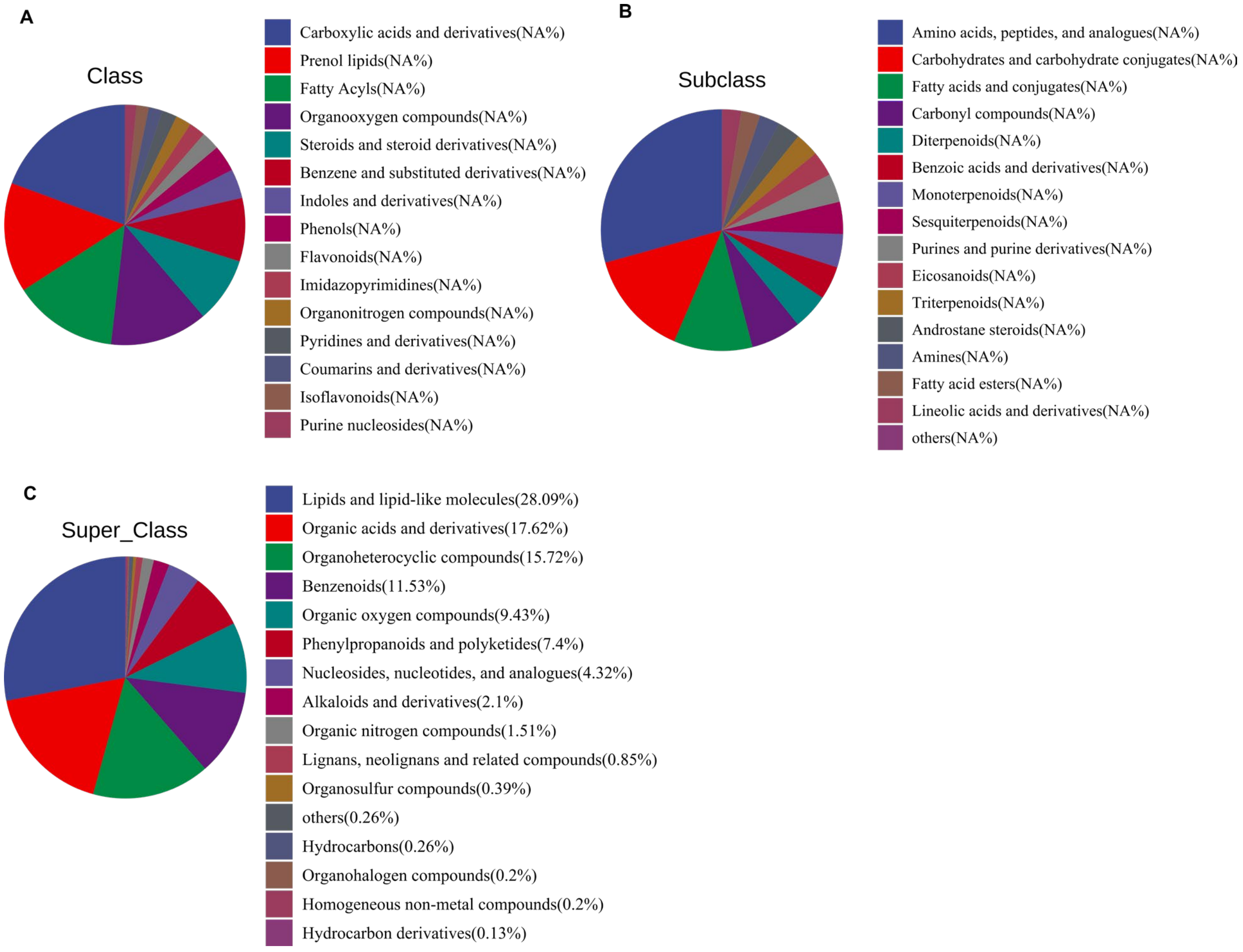

Fig. S2. Comprehensive classification of detected metabolites by chemical structure and metabolic origin. (A) Distribution of metabolites by major class; (B) Subclass categorization; (C) Super-class distribution. "N/A" indicates metabolites detected in the dataset for which chemical superclass or subclass information was unavailable based on the applied annotation database.

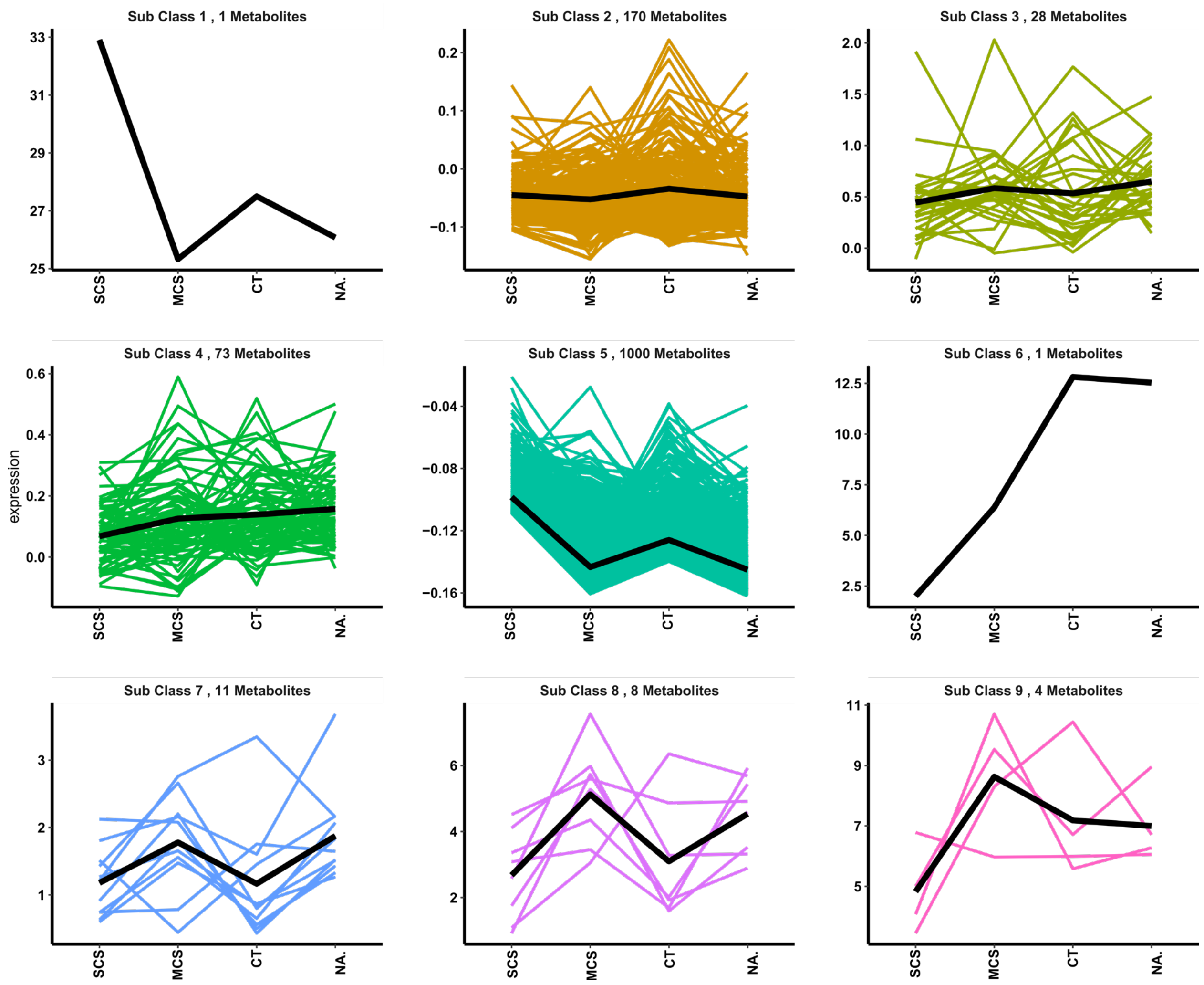

Fig. S3. Dynamic expression patterns of metabolite subclasses across temperature treatments. Each subplot represents a distinct metabolite subclass with individual metabolites shown as separate lines and the bold black line indicating the mean trajectory.

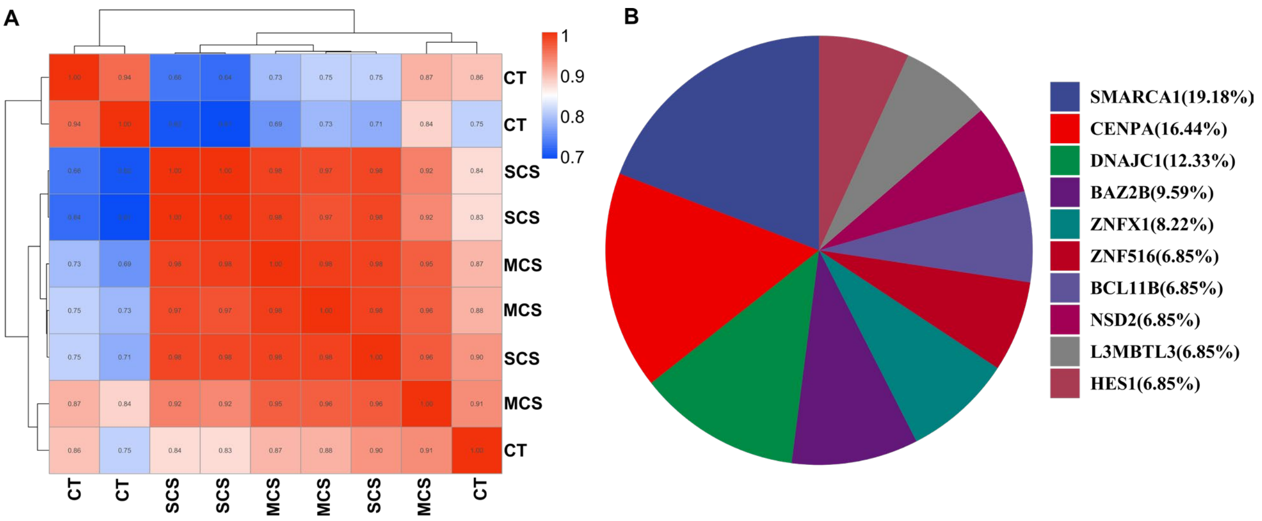

Fig. S4. Transcription factor analysis and sample correlation. (A) Pearson correlation matrix demonstrating high inter-replicate consistency within treatments; (B) Pie-chart showing the distribution of differentially expressed genes annotated to the top 10 transcription factor families.

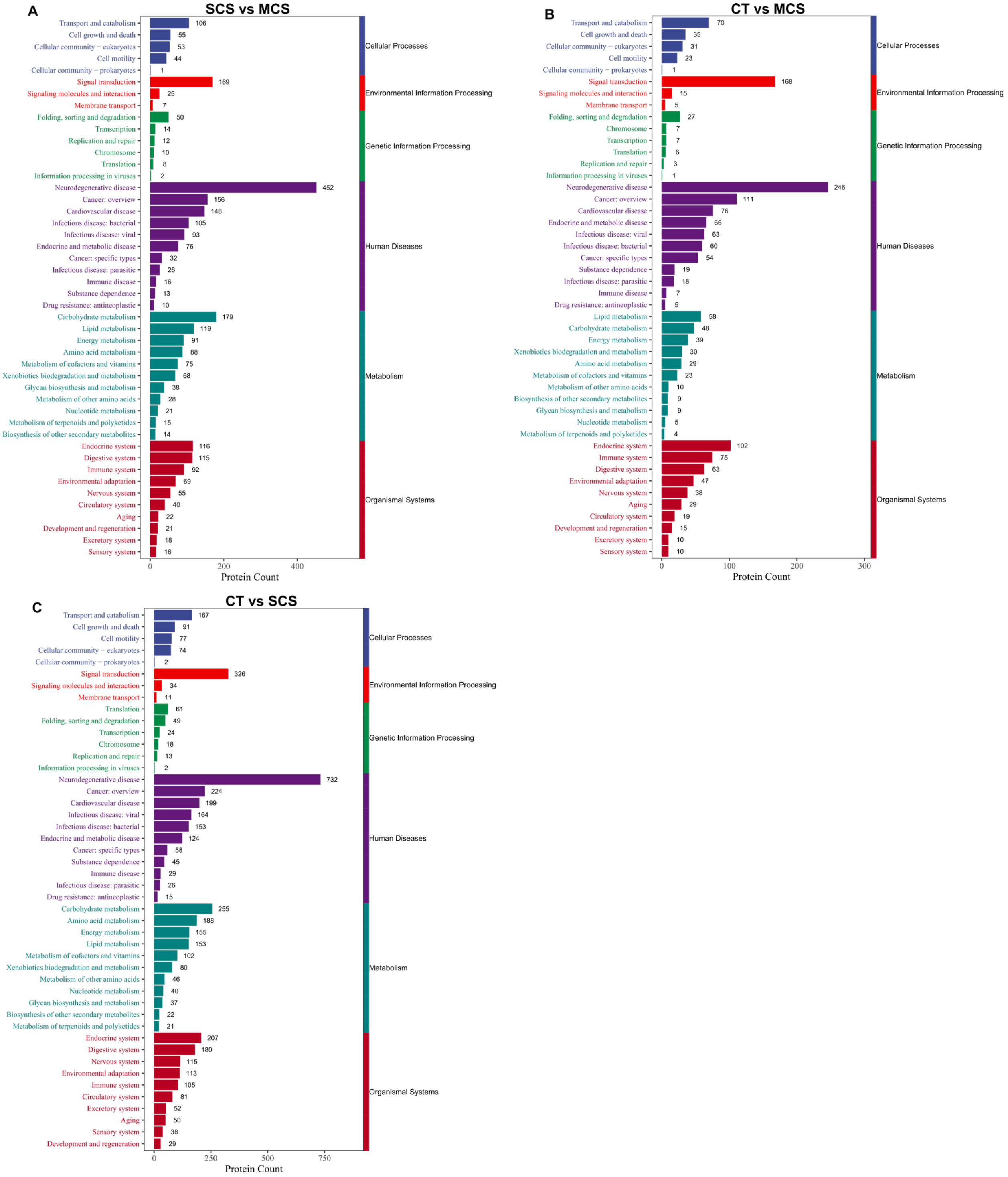

Fig. S5. (A-C) Comprehensive KEGG pathway enrichment analysis highlighting cellular processes, environmental information processing, genetic information processing, metabolism, human diseases and organismal systems categories across all treatment comparisons.

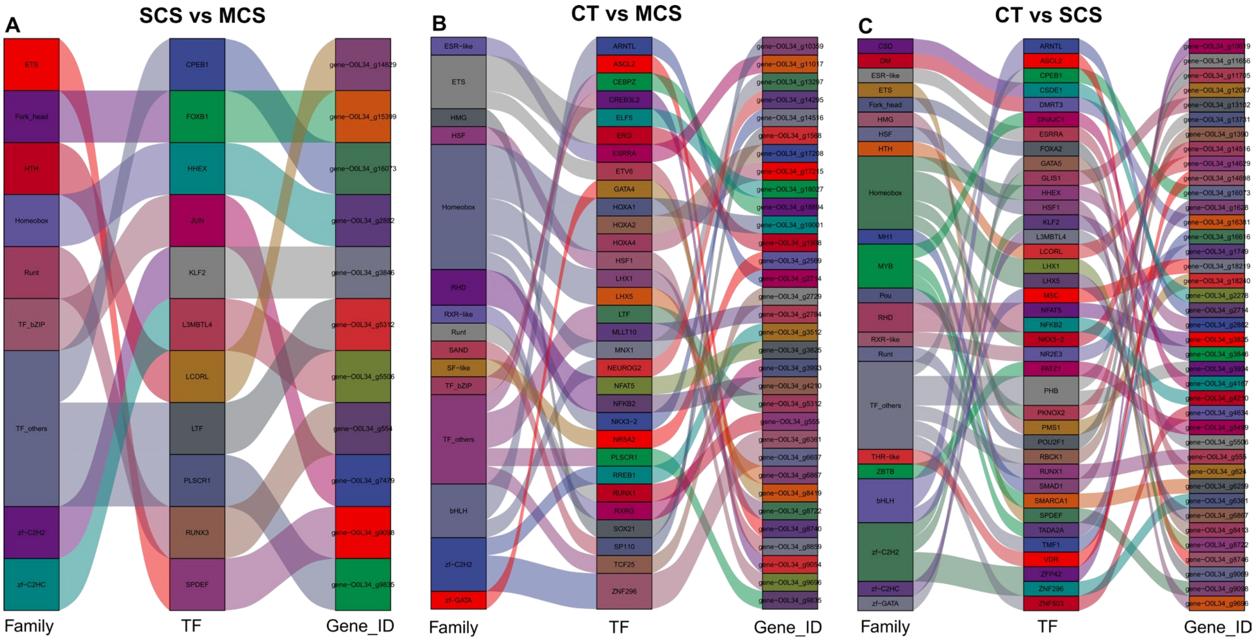

Fig. S6. Gene family-transcription factor-gene regulatory networks. (A-C) Sankey diagrams illustrating the complex regulatory networks connecting gene families), transcription factors and individual genes across treatments comparisons.

Table S1. Forward (*F*) and reverse (*R*) primer sequences used in real-time quantitative PCR

Gene	Primer sequences (5′→3′)	
Caspase-1	F: GTCACTGTCCACGTTCGTCCC<br>R: AACACAAGAACAGGGGCTGGG	[1]
GSTs1	F: ACTTCGAGATTGACCAGAACG<br>R: AGTTTAGCGAGCATTGAGGG	[2]
FOXO	F: GAAATGGAGAGGCAGGGCTGATGTG<br>R: CGCTGTGTAGTGGTGAGTCTGGGAA	[3]
JHAMT	F: GGACCGCTTCATCTCTCCCTACC<br>R: ATTCACCGCTGACACCGCATTTT	[3]
kr-h1	F: CTGGTGAACGACCTTTTGAATG<br>R: GGTATGGTTTTTCTCCTGTGTGTG	[3]
β-actin	F: *CAGGTCCTTACGGATGTC*<br>R: *CTCTTCCAGCCTTCCTTC*	[4]

References

1. Rahmani, S.; Bandani, A.R. Caspase gene silencing affects the growth and development of Tuta absoluta. *Biocatalysis and Agricultural Biotechnology* **2021**, *34*, 102044, doi:https://doi.org/10.1016/j.bcab.2021.102044.
2. Ali, A.; Wu, J.; Xie, J.; Song, Y.; Liu, N.; Liu, X. Functional role of TaGSTs1 in the detoxification of spinetoram and development of Tuta absoluta. *Bull Entomol Res* **2025**, *115*, 754-764, doi:10.1017/s0007485325100321.
3. Wang, X.; Bi, S.; Tang, Y.; Zhang, G.; Huang, C.; Wan, F.; Lü, Z.; Liu, W. Krüppel-homologue 1 regulates the development of Tuta absoluta and its cascade regulation pattern in the juvenile hormone signalling pathway. *Open Biol* **2023**, *13*, 220372, doi:10.1098/rsob.220372.
4. Yang, A.P.; Wang, Y.S.; Huang, C.; Lv, Z.C.; Liu, W.X.; Bi, S.Y.; Wan, F.H.; Wu, Q.; Zhang, G.F. Screening Potential Reference Genes in Tuta absoluta with Real-Time Quantitative PCR Analysis under Different Experimental Conditions. *Genes (Basel)* **2021**, *12*, doi:10.3390/genes12081253.
